# A Fast and Accurate Approach to Multiple-Vehicle Localization and Tracking from Monocular Aerial Images

**DOI:** 10.3390/jimaging7120270

**Published:** 2021-12-08

**Authors:** Daniel Tøttrup, Stinus Lykke Skovgaard, Jonas le Fevre Sejersen, Rui Pimentel de Figueiredo

**Affiliations:** Department of Electrical and Computer Engineering, Aarhus University, Nordre Ringgade, 18000 Aarhus, Denmark

**Keywords:** object detection, multiple object tracking, convolutional neural networks

## Abstract

In this work we present a novel end-to-end solution for tracking objects (i.e., vessels), using video streams from aerial drones, in dynamic maritime environments. Our method relies on deep features, which are learned using realistic simulation data, for robust object detection, segmentation and tracking. Furthermore, we propose the use of rotated bounding-box representations, which are computed by taking advantage of pixel-level object segmentation, for improved tracking accuracy, by reducing erroneous data associations during tracking, when combined with the appearance-based features. A thorough set of experiments and results obtained in a realistic shipyard simulation environment, demonstrate that our method can accurately, and fast detect and track dynamic objects seen from a top-view.

## 1. Introduction

When large vessels such as container ships or ferries are approaching their destination port, they are required by law to have a maritime pilot on board, who is responsible for safely navigating the waters and moving the vessel to its desired location [1]. The maritime pilot has extensive training and knowledge of the local area and knows how the currents and tide affects the vessels in their respective area. However, ensuring safe navigation inside dock environments is demanding as mistakes can be extremely costly, time consuming to correct, or even fatal. Using an unmanned aerial vehicle (UAV) it is possible to anticipate potential dangerous situations and hence help improving safety in the harbor environment. With the use of artificial intelligence (AI) systems, UAVs should be able to detect specific patterns when potentially dangerous situations occur, and provide insights to human operators for aided decision-making to avoid accidents.

This work aims to use UAVs and state-of-the-art computer vision and artificial intelligence algorithms to improve navigation safety and efficiency in harbor environments. We propose a complete solution for an autonomous UAV provided with a small RGB camera attached to the underside pointing straight down to detect and track vessels in harbor environments.

The main contributions of this work are the following:

- a semantic instance segmentation method for reliable and accurate object detection in water environments, using RGB images acquired from aerial images.
- a deep-learning-based tracking approach that can ensure the data association of different objects is robust across frames of a video stream, using an improved bounding-box representation, to allow for reliable multiple object tracking.
- a thorough evaluation of our approaches in a realistic simulation environment.

The rest of this article is organized as follows. First, we overview in detail the state-of-the-art in object detection, segmentation, and tracking methodologies. Then, we present the proposed methodologies for robustly detecting and tracking vessels in harbor environments using RGB images captured from an UAV. In particular, we describe a semantic segmentation-based multi-object-tracking pipeline that extends a state-of-the-art approach, with pixel-level semantic information, for improved rotated bounding-box tracking. Our approach is data-driven, probabilistic and estimates the dynamic state of vessels, through Kalman filtering. We thoroughly describe all the employed methods as well as design decisions during the development of the proposed approach. Finally, we demonstrate the applicability of our methods in a realistic simulation environment, assessing qualitatively and quantitatively our contributions, through a set of experiments, and wrap up with conclusions and suggestions for improvements in future work.

## 2. Related Work

This section outlines the state-of-the-art computer vision techniques to tackle the multiple object-tracking problem, namely object detection, semantic segmentation, and object tracking.

### 2.1. Object Detection

Object detection deals with the problem of determining where objects of interest are in an image (object localization) and which category each detected object belongs to (object classification) [2]. Traditional methods have aimed to solve the object detection problem by in three stages: informative region selection, feature extraction and classification. However, since the groundbreaking work of Krizhevsky et al. [3], AlexNet, a deep convolutional neural network (DCNN) for image classification, achieved record accuracy in the large scale visual recognition challenge (ILSVRC) [4]. In [5] the authors proposed a method for fast object detection method called Fast R-CNN for object detection using CNNs. Their two main contributions to speed up object detection, were to use multi-scale pooling of images, a method called *Spatial Pyramid Pooling* first proposed in [6], and run the neural network once on the whole input image, followed by a Region of Interest (ROI)-Pooling layer that slices the ROI into a fixed size, making it possible to feed an input image of any arbitrary size. In [7], the authors proposed further improvements in a network called Faster R-CNN. The bottleneck in terms of computational efficiency for Fast R-CNN is the region proposal algorithm, which as with the original R-CNN, uses selective search to generate region proposals. Their main contribution is the RPN (Region Proposal Network), a CNN that simultaneously predicts object bounds and objectiveness scores at each position, decreasing region proposals’ computation time.

### 2.2. Semantic Segmentation

**Figure 1:**
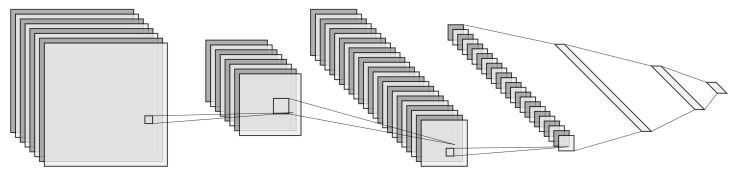
A high-level illustration of a deep learning (DL) object detection network architecture. Layered squares represent feature maps. The connection between stacked feature maps represents convolutional operations. Elongated rectangles to the right represent feature vectors. The connections between feature vectors represent fully connected operations. This figure is created using the tool from [10].

**Figure 2:**
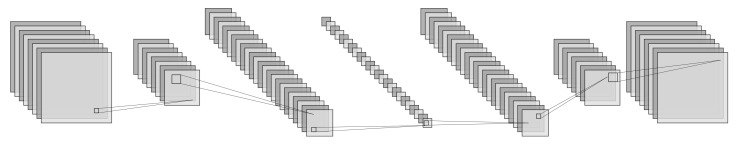
A high-level illustration of a DL semantic segmentation network architecture. Layered squares represent feature maps. The connection between stacked feature maps represents convolutional operations. This figure is created using the tool from [10].

Semantic segmentation is the problem of assigning each pixel in an image to a predefined category [8]. This means the high-level architecture showed in Figure 1 to solve the object detection problem no longer can be applied as the fully connected layers do not preserve the spatial information needed to classify each pixel individually. The fully connected layers are replaced with convolutional layers in the architecture to solve semantic segmentation. In Figure 2 a high-level architecture illustration of a network that solves the problem of semantic segmentation is shown. It can be seen that the network architecture consists of convolutional layers end-to-end. This erases the alignment problem as input to a fully convolutional network (FCN) can have any arbitrary size. Figure 2 represents the overall structure most semantic segmentation network follows. Semantic segmentation networks can mainly be divided into two domains with different end goals: higher accuracy and higher efficiency. Algorithms that address accuracy encompass accurate localization and recognition. These algorithms often have more network parameters and are more computational heavy to better distinguish between object-related categories and make more reliable predictions. Segmentation algorithms that address efficiency focus on efficient convolutional operations such as depthwise separable convolutions [9] and aim for real-time applications.

The architecture of a segmentation algorithm has the same number of dimensional depth in the final output layer as the number of categories the algorithm is trained to detect. The values in the dimensional depth represent a score for each category. This score represents how certain the model is that a pixel belongs to a category.

In the revolutionary paper on fully convolutional networks (FCN) [11] the authors showed a convolutional network, trained end-to-end, pixels-to-pixels, exceeded the state-of-the-art in semantic segmentation. This work was the first to propose a fully convolutional network for pixel prediction which revolutionized the field as future state-of-the-art studies are to a degree an extension of this study. Like prior segmentation models the first part of a fully convolutional network consists of encoding where resolution is reduced, and content is captured. The second part consists of a deconvolution network where resolution is enlarged to recover the lost spatial resolution. To keep the context obtained skip-connections are applied. They fuse layers from the encoding part with corresponding layers in the decoding part. U-Net further exploited the spatial details using dense skip-connections [12]. Another key contribution from this paper is, when using a fully convolutional network, the input size no longer must be fixed as the convolutions no longer are followed by a fixed fully connected neural network.

The fully convolutional network with skip architecture presented in this paper scored significantly better than previous state-of-the-art approaches on semantic segmentation. It scored a relative improvement of 20% over state-of-the-art on the PASCAL VOC 2011 and 2012 test sets, and reduced inference time.

In [13] the authors proposed a network called fast segmentation convolutional neural network. This is a real-time semantic segmentation model on high resolution images suited for embedded devices with low memory and still executes fast computation. The network consists of four different modules: Learning to Down-sample, Global Feature Extractor, Feature Fusion and Classifier. The authors propose some important contributions to the field. A competitive and above real-time semantic segmentation algorithm with 123.5 fps for high resolution images (1024 × 2048 px) and the implementation of skip-connections popular in offline DCNNs with the shallow *learning to downsample* module. The first modules *Learning to Down-sample* objective is to down-sample the feature space, this is efficiently done using depthwise separable convolution first proposed in [9] for reduced computational cost. The next module *Global Feature Extractor* aims to capture the global context for image segmentation. For this is the efficient bottleneck residual block introduced by MobileNet-V2 [14] used where the bottleneck blocks uses depthwise separable convolutions resulting in fewer model parameters. The third module *Feature Fusion* fuse the output of the Learning to Down-sample module with the output of Global Feature Extractor to fuse global context with skip-connections to recover lost resolution. The last module *Classifier* upsample the feature space to match the spatial size of the input image. The Fast SCNN network was compared with other state-of-the-art real-time semantic segmentation methods and showed improved state-of-the-art runtime with minor loss in accuracy.

### 2.3. Multiple Object Tracking

The problem of detecting and associating objects across frames, maintaining their identities, and estimating their individual trajectories over time is called Multiple Object Tracking (MOT). In this work, we use the SORT algorithm [15], a widely used MOT technique that performs Kalman filtering in image space, and frame-by-frame data association using the Hungarian method with a simple association metric that measures bounding-box overlap. A later extension called DeepSORT [15] enables the framework to track objects through longer periods of occlusions, thus reducing the number of identity switches using robust appearance-based features learned with deep learning methods. To minimize the inference time, much of the computational complexity has been placed into an offline pre-training stage to learn a deep association metric on a re-identification dataset. The recent work of [16] proposes a method called Track R-CNN which jointly addresses detection, tracking and segmentation, claiming that box-level tracking performance improvements may only be possible when moving to pixel-level classification. Track R-CNN extends Mask R-CNN with 3D convolutions to incorporate temporal information and data association (i.e., re-identification) to link object identities over time, in a single forward pass.

## 3. Methodology

In this section, we overview in detail the pipeline proposed for multiple object detection and tracking estimation between vehicles seen from aerial images. Furthermore, we introduce the method for obtaining the data set used to train and validate our methodologies.

A brief explanation of the input, output and role of each system stage is outlined below.

- **Object detection** takes RGB images as input and is responsible for detecting object instances in each image. Outputs a list of axis-aligned bounding boxes, and rotated bounding boxes.
- **Multiple object tracking** Takes a list of axis-aligned bounding boxes as input and tracks objects across frames, to estimate the orientation and velocity of the objects, the velocity vectors of each tracked object

### 3.1. Data Collection Method and Tools

**Figure 3:**
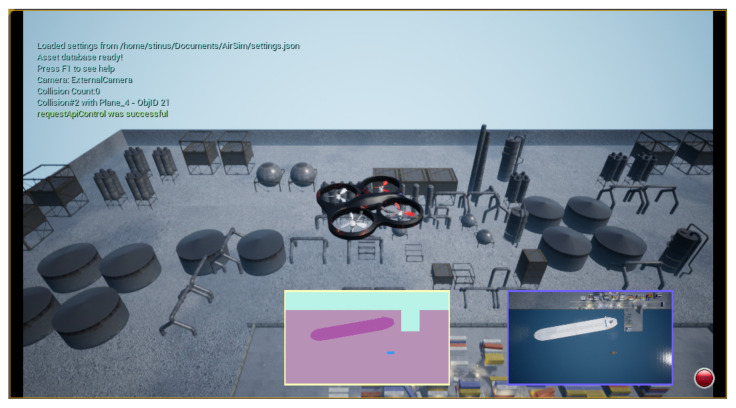
Interface of Airsim in unreal engine.

The dataset used in this work has been collected using the popular game engine called Unreal Engine 4. This game engine is popular inside the field of robotics as Microsoft has made a plugin for the engine that allows for drone simulation. The plugin is called AirSim [17]. It allows for the collection of both RGB images alongside annotated ground-truth images. On Figure 3 a screenshot from the Airsim interface can be seen. There are two additional screens inside the main view, showing a live feed of the downward-facing camera. The right screen shows the RGB image, while the left one shows an annotated image feed. Different color inside the annotated view corresponds to different object categories. On Figure 3 it shows the categories land, tugboat, large vessel, and water.

The annotated images are used as the ground truth. They are pixel-accurate because the image is obtained in simulation, making each object model possible to label in AirSim. This has the benefit of not only being accurate but also more convenient than manually label images, making it relatively easy to generate datasets of a decent size.

A dataset has been created for training the detector model and will be described in detail in Section 5. The training dataset characteristics are:

- **Dataset size:** 1565 RGB images and 1565 annotated ground-truth images
- **Image size:** Both the RGB and annotated images are 640 × 360 pixels with three color channels
- **Large vessel class:** 1058 instances of large vessel in the dataset
- **Tugboat class:** 3580 instances of tugboats in the dataset
- **Land class:** 1537 instances of land

The test dataset used has the same sized images of 640 × 360 pixels and the same number of color channels. The number of images is 198, and the class balance is as follows:

- **Large vessel:** 194 instances of large vessel
- **Tugboat class:** 274 instances of tugboat
- **Land class:** 168 instances of land

**Figure 4:**
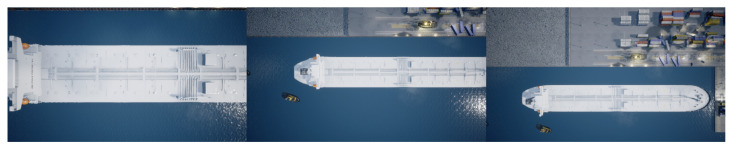
Images from the simulation environment captured at different altitudes.

It is possible for multiple instances of a class to be present in a single image, as seen on both datasets. This is especially apparent with the tugboat class in both datasets. Even though more instances of tugboats are present in the images, the number of images with each class present is close to the same. The images are a mix of classes taken at different altitudes and always pointing straight down from the UAV. Examples from the datasets can be seen on Figure 4.

The used AirSim environment has been made to resemble a real-life harbor environment. When training a model on images obtained in a simulation environment, it is essential to make the simulation data resemble real-life images to bridge the gap between simulation and real-life. However, bridging the gap between simulation and real-life is not the focus of this work since only simulated images will be used for training and testing.

Considering the realistic rendering engine used in our experiments, the simulation-reality gap should be easily overcome with a few real-life samples, as demonstrated in [18,19].

### 3.2. Design Decisions and Considerations

In this section, the design decision and consideration for the object detection stage. Different solutions will be discussed and compared, leading to a final solution for implementation.

**Figure 5:**
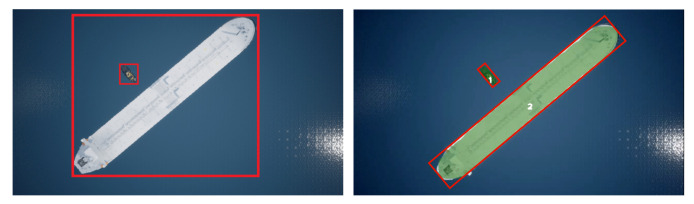
(**left**) Worst case scenario with axis-aligned bounding boxes. The bounding box of the small ship is inside the bounding box of the large ship. (**right**) Segmentation mask superimposed on top of the two ships. A rotated bounding box is made based on segmentation, thus representing the vessel’s spatial location more precisely.

A vessel’s shape would be highly misrepresented by axis-aligned bounding boxes, if it is located diagonally in the image plane (see Figure 5).

To better represent the shape and size of each vessel, algorithms such as Fast SCNN [7], and U-Net [12] have been considered. These provide a pixel-level representation of objects as they perform semantic segmentation. Compared to the object detection algorithms, this would produce a very accurate representation of vessels. The segmentation output can be used to produce a rotated bounding box that is fitted to the segmentation mask (see Figure 5) that better encloses the vessel shape.

**Figure 6:**
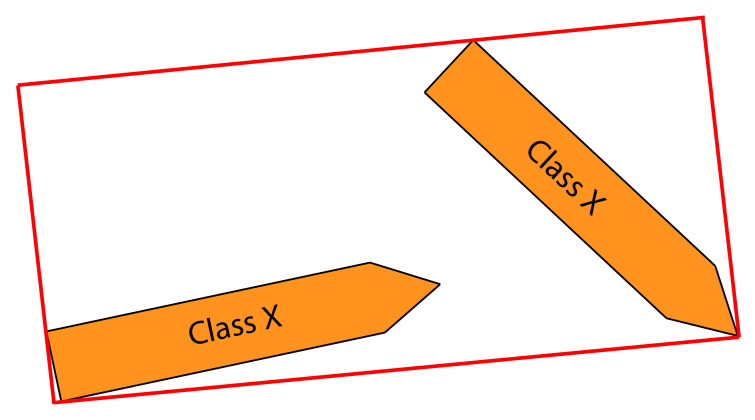
Rotated bounding box encompassing two instances of the same class.

For this bounding-box representation to be possible, a method for fitting a rectangle to the segmentation mask is needed. Fortunately, such a method proposed by Godfried Toussaint called rotating calipers [20]. This algorithm will be explained in detail in Section 3.3.4. However, there are still situations where semantic segmentation is not enough. Semantic segmentation is not sufficient in situations where multiple instances of the same class are present in the image simultaneously, as semantic segmentation does not discriminate between object instances of the same class. On Figure 6 it can be seen how object instances of the same class are not discriminated against and affect the bounding-box representation. Based on this limitation, it was determined to go for an instance-based semantic segmentation algorithm.

Instance-based semantic segmentation algorithms overcome the issue of multiple instances of the same object class. A number of different instance-based semantic segmentation algorithms have been considered in this work. These methods are MaskR-CNN, TrackR-CNN, and YOLACT.The quality of the rotated bounding boxes is highly dependent on the model’s ability to produce accurate and reliable segmentation, as deviations in the segmentation will be directly translated into the rotated bounding boxes’ shape. For this reason, was YOLACT discarded as it produces lower accuracy results compared to MaskR-CNN and TracR-CNN. TrackR-CNN is capable of more than Mask R-CNN as it also includes tracking while simultaneously providing instance-based semantic segmentation. However, this comes at the cost of being more computationally heavy than MaskR-CNN. TrackR-CNN uses 3D convolutions to include the temporal element of tracking objects from frame to frame. According to [21], the computational complexity of 3D convolutions can be expressed as in

(1) $$C_{o}\times C_{i}\times T\times H\times W\times K_{T}\times K_{H}\times K_{W}$$

where $C_{0}$ and $C_{i}$ are the input and output channels, *T*, *H*, *W* represents the time (or the number of frames), height, and width, respectively. $K_{T}$, $K_{H}$, $K_{W}$ is the temporal dimension, height, and width of the kernel. For 2D convolutions, the complexity can be expressed as in

(2) $$C_{o}\times C_{i}\times T\times H\times W\times 1\times K_{H}\times K_{W}$$

It can be seen how the temporal dimension of the kernel is 1, meaning that 2D convolutions do not take previous or future frames into account. Because of this added computational complexity of 3D convolutions, TrackR-CNN was discarded, as it was believed that the combination of MaskR-CNN and a real-time tracking algorithm would be faster in terms of inference time. More design considerations about the tracker will be discussed in Section 4.

### 3.3. Chosen Solution in Detail

This section covers Mask R-CNN in detail, together with the procedures of creating rotated bounding boxes and final detection filtering.

**Figure 7:**
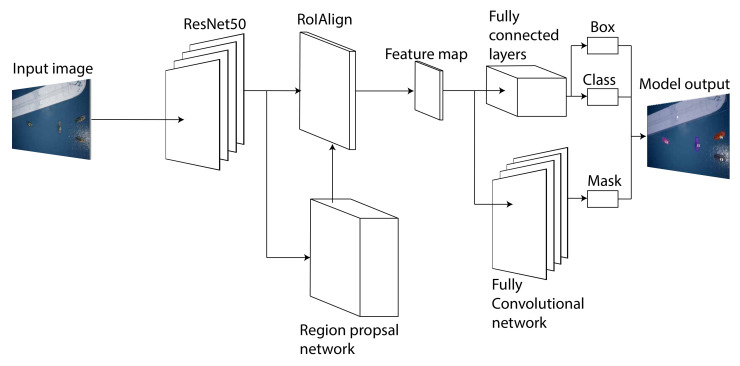
High-level illustration of the architecture of Mask R-CNN.

As discussed in the previous section, Mask R-CNN [22] is selected as the detector for this work. A detailed rundown of how this detector works will be discussed in this section. Mask R-CNN builds upon the popular Faster R-CNN architecture by expanding it with a segmentation branch and an improved region of interest (RoI) pooling module called RoI Align. A high-level illustration of the different components of Mask R-CNN can be seen on Figure 7. This shows the exact architecture of the specific implementation of Mask R-CNN that has been used in this work.

As it can be seen on Figure 7 the backbone network used is ResNet50 [23]. As the name suggests, it is a ResNet network with 50 layers. The backbone network has the responsibility of generating a feature map that is fed into both the region proposal network (RPN) and RoI Align module. The RPN has the responsibility of generating RoIs. The RPN and RoI Align module were first introduced in Faster R-CNN [7] network and improves the inference time of Faster R-CNN compared to its predecessor. The RoI Align module outputs a fixed-size feature map for each RoI proposed by the RPN. These feature maps are passed to two separate branches: a sequence of fully connected layers and a sequence of convolutional layers. The fully connected layers eventually branch out into two output layers where one provides the classification scores, and the other outputs the four coordinates for each bounding box. The convolutional layers predict the segmentation mask for each feature map provided by the RoI Align module.

#### 3.3.1. Region Proposal Network

The RPN aims to predict region proposals with different scales and sizes. It cuts regions from the feature map provided by the backbone network. These regions are produced by generating a large number of anchors, which essentially are rectangles of different sizes and aspect ratios. During training, the RPN is trained to find regions based on ground-truth bounding boxes provided in the training dataset. The training is based on the scores obtained from its classification branch score and box regression branch score, as these are used inside the RPN. At test time, the RPN outputs the top N performing regions based on the classification score, which is then passed to the RoI Align module. The coordinates of the regions are based on the original image, and deviates in scales and aspect ratios. Because the last part of Mask R-CNN consists of fully connected layers, there is a need to align the proposed regions to a fixed size, as the RoI Align module does this task.

#### 3.3.2. RoI Align Module

The input to the RoI Align module is the RoI coordinates and the feature map generated by the backbone network. Because the coordinates of each region are made w.r.t the original image, the region proposal needs to be aligned to the feature map. Using bi-linear interpolation, it is possible to output a feature map of a fixed size no matter the size of the input feature map.

#### 3.3.3. Object Detection and Segmentation Branch

After the RoI Align module, the bounding boxes are passed through a set of fully connected layers, which outputs the probability of the classes and coordinates for the bounding boxes. The output from the RoI Align module is also passed to the mask branch, which uses an extra fully convolutional network to output a probability map of the bounding boxes.

**Figure 8:**
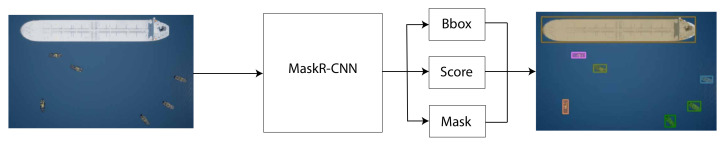
A high-level illustration of input and output of Mask R-CNN.

The output from Mask R-CNN is shown on Figure 8. The transparent colors represent the mask corresponding to each object. The colors are not outputted from Mask R-CNN but chosen randomly to showcase its instance semantic segmentation capabilities. Furthermore, the bounding boxes are also shown.

As discussed in Section 3.2 the output from the detector needs to be further processed to produces rotated bounding boxes. Furthermore, filtering of redundant detections will be accomplished using non-maximum suppresion (NMS). These two concepts will be discussed in the following two sections.

#### 3.3.4. Rotating Axis-Aligned Bounding Boxes

**Figure 9:**
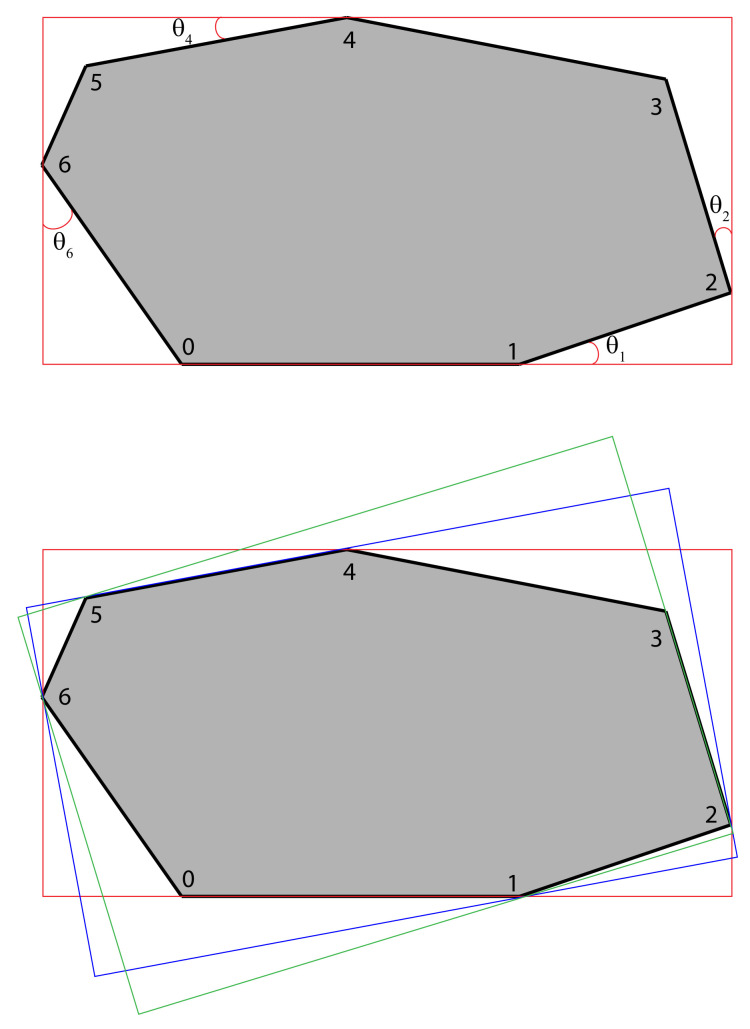
**Top**: Initial bounding-box encompassing a polygon. **Bottom**: 3 rotations of the “calipers”.

To obtain a better bounding-box representation of the detected objects, an algorithm called “rotating calipers” [20] is used. It is an algorithm that is highly efficient at producing the smallest-area enclosing rectangle with a computational complexity of O(n). The algorithm works by rotating a set of orthogonal support lines around a polygon. These lines can be thought of as a set of calipers rotating around a polygon once. In this work, the open-source library OpenCV [24] is used to find the rotated minimum fit rectangle to enclose each objects’ segmentation mask, which is based on this algorithm (see Figure 9).

Before this algorithm is applied to a segmentation mask in the object detection stage, some pre-processing is needed. Each segmentation mask is converted into a contour, which is an outline representing the shape of the bounding box. Using this contour, the rotating calipers algorithm is applied which produces the rotated bounding boxes.

The algorithm first aligns a bounding box to have a coincident edge with a polygon edge. This can be seen on Section 3.3.4 where the red bounding box lies on the edge between vertex 0 and 1. The next step is to find where the minimum angle between the red bounding box and the convex hull is. For the example on Section 3.3.4 this is $\theta_{4}$.

Then the box is rotated by $\theta_{4}$ making the rotated box coincide on edge between vertex 4 and 5. The box is either enlarged or shrunk after each rotation to encompass the entire shape of the polygon. When the rotation is done, the area is computed, replacing a previous computed area if it is smaller, and another rotation is done. Multiple rotation steps can be seen on Section 3.3.4. When the rotation reaches $360^{\circ }$, the rectangle with the smallest area is left and used as the optimized rotated bounding box.

#### 3.3.5. Non-Maximum Suppression

Before feeding the axis-aligned bounding boxes to the multiple object tracking (MOT) stage, some final filtering of detections is done. This is to filter out detections with a low confidence score using a confidence score threshold. To avoid overlapping bounding boxes non-maximum suppression is used. Non-maximum suppression is a key post-processing method used to avoid multiple bounding boxes for the same object. It works by going through each detection, starting with the detection with the highest confidence score, and compute the bounding box intersection over union (IoU) with the other detections bounding boxes. If the IoU is above a given threshold, the bounding box with the lowest score is suppressed. Because this system operates with 2D images taken by an UAV, it is assumed that objects do not overlap, therefore, the IoU threshold overlap is set to 0.1. The rotated bounding boxes presented in Section 3.2 are used for non-maximum suppression as this gives a much better representation of the object and allows for having a low IoU threshold without wrongfully suppressing wanted bounding boxes.

On Figure 5 in Section 3.2 shows an example of two vessels with axis-aligned bounding boxes. If non-maximum suppression is done to that scenario, it would remove the bounding box of the vessels with the lowest confidence score, which is an unwanted behavior. However, if instead rotated bounding boxes, as on Figure 6, are used for non-maximum suppression, none of the bounding boxes would be unconsidered.

### 3.4. Training Mask R-CNN

**Figure 10:**
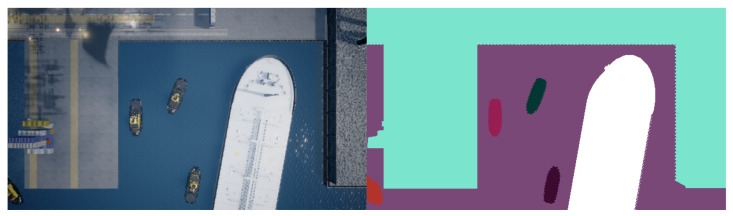
(**Left**) Raw image. (**Right**) Annotated ground-truth image.

To optimally train the Mask R-CNN network, precise ground-truth labels for the training data are needed. In this work, the images are, as previously mentioned, provided by Unreal Engine with the AirSim plugin. This makes it possible to obtain annotated ground-truth images alongside the raw images. In Figure 10 an example of what both the raw image and the annotated image resemble is shown.

**Figure 11:**
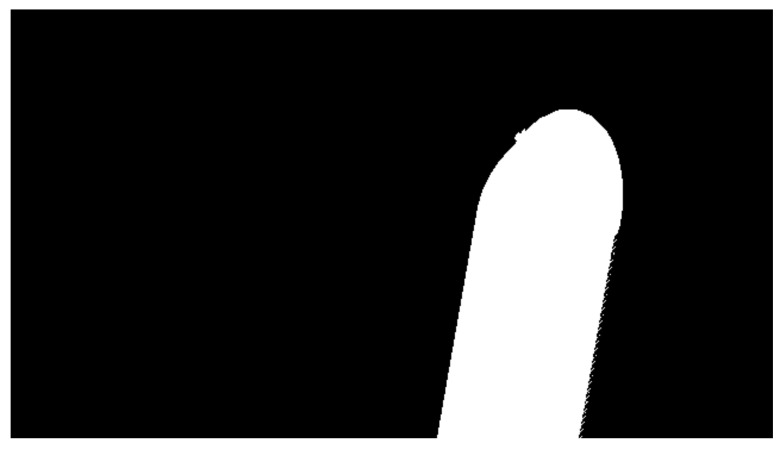
Binary mask of a single object instance.

It can be seen how each category is labeled with a different color on Figure 10. The land category has a turquoise color, the large vessel has a white color, the water is purple, and the tugboats have different colors. Multiple instances of the same category must be colored uniquely and still maintain the same category label for the Mask R-CNN network to distinguish between different instances. In the process of generating box coordinates of all object instances, the annotated image is transformed into a set of binary masks, with each binary mask representing an object instance. A binary mask is represented as a matrix with a shape of m×n, *m* being the width and *n* being the height of the image. The color channels are discarded, and the location of the object by the value (see Figure 11).

The final step of generating the bounding boxes is to find the minimum and maximum on the x-axis alongside the minimum and maximum of the y-axis. This yields the (x,y) coordinates of the top-left and bottom-right corners that can be used as a bounding box to encompass the entire object. The same procedure is done for all the binary masks. Afterward, they are all labeled with their respective category number (1 for a large vessel, 2 for a tugboat, and 3 for land). Their mask is saved in a list with all this information ready to be used for training.

## 4. Multiple Object Tracking

This section covers the multiple object-tracking stage methodologies. It will involve the challenges of MOT and how these challenges are being solved in state-of-the-art solutions. Next, design decisions and considerations are detailed. Lastly, a thorough explanation of the chosen solution of the MOT stage is presented.

### 4.1. Brief Review of MOT

MOT is another fundamental computer vision problem. Like other computer vision challenges, MOT has become a research hotspot with the rapid development of deep neural network (DNN). According to [25], the task of MOT is predominantly partitioned into locating multiple objects, maintaining their identities, and yielding their trajectories. The rapid development in DNN has significantly improved the accuracy of object detection. As a result, the framework tracking-by-detection has become the most popular framework to solve the challenge of MOT.

**Figure 12:**
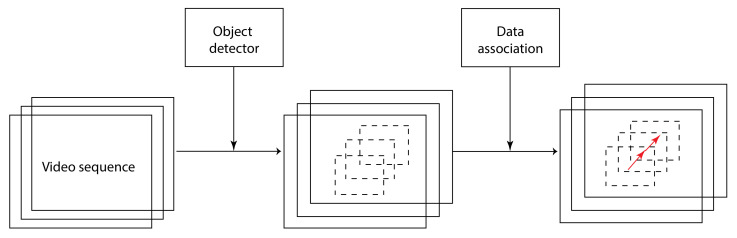
High-level illustration of tracking-by-detection framework.

The tracking-by-detection framework works by decoupling the task of the detector and the tracker. The task of the detector is to detect all objects in each frame. These detected objects are then fed to the tracker. The tracker performs data association on the detected objects across frames to obtain their trajectories. Therefore, the data association problem is a key step to overcome in a tracking-by-detection framework. As the tracking algorithm relies on detections from the detector to locate objects in each frame, the tracker’s performance largely depends on the quality of the detector. On Figure 12 is a high-level illustration of the tracking-by-detection framework.

Data association is the process of associating uncertain detections to known tracks. The typical data association procedure is first to make a new detection, then from the predicted tracks yield a space where the detection is expected. This space is also called a validation gate. Lastly, there is a match if the new measurement lies within the validation gate space.

The problem of data association has been solved in numerous ways. One of the simplest ways is used in [26], in which the authors propose to solve the data association problem by associating detections across frames using their spatial overlap as validation gate. Because of the aforementioned significant increase in object detectors’ quality, straightforward tracking algorithms such as the IoU tracker can achieve good results. Furthermore, because they are simple, data association can be done at a high frame rate. However, if challenging scenarios such as camera motion, occlusion of objects, or crowded scenes occur, simple data association methods fall through, and more complex algorithms are needed.

Hungarian method, also known as Kuhn–Munkers algorithm [27] is another used method to perform data association in the MOT problem. The algorithm was first introduced to solve the assignment problem in polynomial time. When the algorithm is applied to the MOT problem, it is explained as finding the optimal matching solution for several targets. This makes it possible to pair new detections to tracks based on a predefined score. Examples of scores are the overlap of bounding boxes across frames or the cosine distance between feature vectors generated by a convolutional neural network (CNN). An assignment cost matrix is then computed based on the score, and matching is done by solving the assignment problem optimally with the Hungarian algorithm.

MOT algorithms can be divided into two different categories: Offline methods and online methods. Offline methods, also called batch tracking algorithms have access to future information (i.e., future frames) when associating detections across frames. Often no inference time requirements restrict these methods allowing for more complex solutions yielding better overall performance. Online tracking algorithms, on the contrary, only have access to current and past information to make predictions in the current frame. Another important reason online tracking algorithms perform worse is that they cannot fix past errors by looking into future frames. Even though online tracking algorithms only use past and present information, they do not necessarily run in real time. Often, they are too slow to run on real-time equipment. Therefore, online real-time tracking algorithms are required to be simple and avoid complex computations to achieve real-time inference requirements [28].

### 4.2. Design Decisions and Considerations

This section provides an overview of the considerations and design decisions for the MOT stage. Different solutions will be discussed and compared, leading to a final solution for implementation.

The input to the MOT stage is the axis-aligned bounding boxes outputted by the object detection stage. The output of the MOT stage is a velocity vector of each tracked object. This is the black box representation of inputs and outputs of the tracking stage.

Two main tasks can be derived for the MOT stage to complete:

- **Task 1** Perform data association of detections across frames.
- **Task 2** Output velocity vector of each tracked detection.

Task 1 of the MOT stage is to overcome the data association problem described in the previous section. This system is aimed to run on a UAV with limited computational power in real time. Consequently, the tracking algorithm must compute and run the tracking online, with low inference time. The camera is attached to the underside of the UAV making the tracking problem less complex as it can be assumed objects in the image cannot occlude each other. This is a crucial assumption because 3D scenes require a more complex tracking network to solve the task of MOT.

The increasing research attention on MOT has lately boosted the number of MOT algorithms that follow the tracking-by-detection framework, making it impossible to investigate and evaluate all tracking algorithms. Therefore, has a small number of algorithms been chosen for comparison in the following. For this work, TrackR-CNN [16], SORT [15], Deep SORT [29] and Flow-Tracker [30] have been considered, each with their own pros and cons. All these four contenders are tracking algorithms by the tracking-by-detection framework and can work in continuation of the object detection stage.

As mentioned in the Section 3.2 were the TrackR-CNN considered to be a network for handling both instance-based semantic segmentation and object tracking as an extension of Mask R-CNN using 3D convolutions. This network was discarded because of the unnecessary complexity introduced by 3D-convolution layers. In [16] the authors presented a high inference time resulting in 2 frames per second (FPS) on a Titan X (Pascal), with an extensive need for memory as three images are fed in at once. This makes the TrackR-CNN unsuited for real-time tracking.

Both SORT [15] and Deep SORT [29] were considered to solve the MOT problem. Deep SORT is an extension of SORT, and the architecture of the two algorithms is much alike. Both algorithms are based on Kalman filter and the Hungarian algorithm to solve the data association problem. SORT is the simpler one and uses the IoU distance between each detection and all predicted bounding boxes from existing targets as an assignment problem solved by the Hungarian algorithm. Deep SORT instead uses a small CNN to extract feature vectors from each detection and calculate the smallest cosine distance fused with the Mahalanobis distance between predicted Kalman states and detections as an assignment problem solved by the Hungarian algorithm. This difference makes the Deep SORT better at handling occlusions and objects leaving and re-entering the scene without ID switches, but it comes with a computational cost. The authors ran both tracking algorithms on a GTX 1050 mobile GPU and achieved 60 fps for SORT and 40 fps for Deep SORT, with a significant decrease in ID switches for Deep SORT. With both tracking algorithms well suited for online real-time tracking and Deep SORT achieving better tracking accuracy, Deep SORT is a better contender for a tracking algorithm in this work. Another advantage of using a tracking algorithm based on the Kalman filter is the state estimation. A velocity vector of the tracked objects can be derived from the Kalman filter, to achieve task 2 listed above.

The Flow-Tracker proposed in [30] is another MOT tracker following the tracking-by-detection scheme. The Flow-Tacker uses an IoU Tracker as a baseline because of its simplicity and high efficiency. The simplicity and high efficiency come at a cost as camera motion leads to many errors and ID switches. The authors accommodate for this by eliminating the effect of camera motion by estimating the motion between two adjacent frames. The method also uses a small CNN to extract feature vectors of bounding boxes to minimize the number of ID switches. Flow-Tracker was tested on a GTX1080Ti GPU and scored a speed of 5 fps. Even though this tracker scores high on average accuracy and precision compared to Deep SORT, the tracker’s inference time is too high to be considered a real-time tracker and therefore discarded. Deep SORT is chosen in this work, for its accuracy and real-time capabilities. Furthermore, Deep SORT uses a Kalman filter which enables easy extraction of each object’s velocity vector.

### 4.3. Chosen Solution in Detail

To perform MOT the deep learning-based tracking algorithm, Deep SORT [29] is used. Deep SORT is based on a frame-by-frame data association method and Kalman filtering. This method is aimed at tracking scenarios where the camera is not calibrated, and no ego-motion of the camera is available.

**Figure 13:**
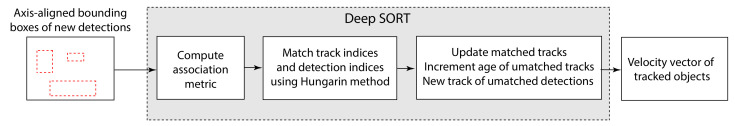
High-level pipeline illustration of how Deep SORT is used in the proposed solution.

On Figure 13 is a simplified pipeline of how the Deep SORT algorithm is integrated into the proposed solution. The detector feeds axis-aligned bounding boxes corresponding to new detections to the tracker. This step is illustrated on the first box of Figure 13.

Deep SORT uses the Hungarian algorithm, to solve the data association problem. The first step within Deep SORT is to compute the association metric, which is a fusion of motion and appearance information.

The motion information is measured as the Mahalanobis distance between the predicted Kalman states and the newly measured states. Camera motion or occlusion of the camera’s viewpoint can introduce rapid and unpredictable changes in the placement of objects from frame to frame, making the Mahalanobis distance metric uninformed for tracking. Therefore, an additional metric is introduced to the assignment problem solved by the Hungarian method. For each bounding box, an appearance descriptor is computed by a pre-trained CNN. This CNN consists of two convolution layers, one max-pooling layer, six residual block layers, and at last, a dense, fully connected layer resulting in a feature vector of size 128. The second metric is calculated as the smallest cosine distance between the tacked and the newly generated feature vectors obtained from new detections. The CNN has been trained on 40 instances of each class. These two metrics are fused into one by a weighted sum

(3) $$c_{i,j}=\lambda d^{\left(1\right)}\left(i,j\right)+\left(1-\lambda \right)d^{\left(2\right)}\left(i,j\right)$$

where $c_{i,j}$ is the weighted sum metric of *i*-th track and *j*-th bounding box, λ is a hyper parameter, $d^{\left(1\right)}\left(i,j\right)$ and $d^{\left(2\right)}\left(i,j\right)$ is the motion metric and appearance metric. This metric is used to solve the association problem with the Hungarian method (the second block within Deep SORT in Figure 13).

The output of the Hungarian algorithm is either a match between a track and a new bounding box, an unmatched track, or an unmatched bounding box. If it is a match between a track and a new bounding box, the track will be updated with new information, and the age of the track be reset to 0. If it is an unmatched track, the age of the track is incremented by one. If it is an unmatched detection, a new track is made. The age of a track makes sure it is not deleted if the track is not matched with a new detection for a couple of frames. This can happen if an object is occluded or leaves the viewpoint of the camera.

Track handling and the Kalman filtering framework are an integral part of Deep SORT. How Deep SORT defines the tracking scenario and performs track state updates is explained in detail. Bounding boxes fed to the Deep SORT algorithm has the format (u,v,γ,h), where (u,v) is the center coordinate of the bounding box, γ is the aspect ratio, and *h* is the height of the bounding box.

Deep SORT describes the tracking scenario as an eight-dimensional state space $\left(u,v,\gamma ,h,\hat{x},\hat{y},\hat{\gamma },\hat{h}\right)$. $\left(\hat{x},\hat{y},\hat{\gamma },\hat{h}\right)$ is the velocity in *x*, *y* direction and velocity in aspect ratio and height. Deep SORT uses a standard Kalman filter with constant velocity and take bounding-box coordinates (u,v,γ,h) as direct observations for the object state.

The Kalman filter updates a track’s state if a match between a new detection and a track is made. This is done using the estimated state of the track from the previous time step and the measured state of the detection at the current time step to estimate the current state of the track. This makes the Kalman filter a recursive estimator. The Kalman filter equations can be written in a single equation, but it is usually explained in two distinct phases: the predict and the update phase. The predict phase produces an estimate of the current state of an object using the state estimate of the object at the previous time step. In this context, the predict phase will estimate the current state of a track using the estimated state of the track at the previous frame. Equations (4) and (5) are two equations to explain the predict phase of the Kalman filter.

(4) $${\hat{x}}_{k}=F_{k}{\hat{x}}_{k-1}$$

where ${\hat{x}}_{k}$ is the estimated state of the track at frame k, $F_{k}$ is the state transformation at frame k describing how the track propagates through time, ${\hat{x}}_{k-1}$ is the estimated state of the track at frame *k*−1.

(5) $$P_{k}=F_{k}P_{k-1}F_{k}^{T}+Q_{k}$$

$P_{k}$ is the estimated covariance at frame k and explains the uncertainty of state parameters. State parameter are (u,v,γ,h). ${\hat{x}}_{k}$ are used as the predicted state when calculating the Mahalanobis distance between the predicted state of a track and the measured state of new detection.

The update phase calculates the residual difference in the current prediction state of the track with the current measured state of the track. On Equations (6)–(10) are the equations to explain the update phase listed.

(6) $${\tilde{y}}_{k}=z_{k}-H_{k}{\hat{x}}_{k}$$

${\tilde{y}}_{k}$ is the measurement pre-fit residual. It calculates the mean error between the observed position of the track and the estimated position of the track. $z_{k}$ is the track position at the current frame k. $H_{k}$ is called a measurement matrix and are ones on the diagonal where the observations are located.

(7) $$S_{k}=H_{k}P_{k}H_{k}^{T}+R_{k}$$

$S_{k}$ is the pre-fit residual covariance, $R_{k}$ is a noise matrix of the track. It is a 4 × 4 diagonal matrix with the noise of the center point (u,v), width, and height as values, according to:

(8) $$K_{k}=P_{k}H_{k}^{T}S_{k}^{-1}$$

$K_{k}$ is the Kalman gain. The Kalman gain is a fusion of the uncertainty in the state parameters $P_{k}$ and the uncertainty in the observations $S_{k}$.

(9) $${\hat{x}}_{k}={\hat{x}}_{k}+K_{k}{\tilde{y}}_{k}$$

${\hat{x}}_{k}$ is the enhanced estimation of the estimated state at the current frame k using the Kalman gain $K_{k}$ together with the measurement pre-fit residual ${\tilde{y}}_{k}$ and add it to the estimated ${\hat{x}}_{k}$

(10) $$P_{k}=\left(I-K_{k}H_{k}\right)P_{k}$$

This is the final step of the update phase. $P_{k}$ is the updated covariance matrix at the current frame k. It is calculated by Kalman gain $K_{k}$ and the estimate covariance $P_{k}$

After all states of the newly matched tracks have been updated, state parameters $\hat{x}$ and $\hat{y}$ that describes the velocity of a tracked object in x and y direction are derived and used as the output of the MOT stage.

We note that for the sake of simplicity, in the current proposed solution only the vessels’ motion is modeled, and self-motion unconsidered. However, self-motion could be easily introduced by accounting for inertial and absolute measurement sensors placed on the UAV (e.g., GPS, IMU, altimeter).

## 5. Results

This section covers all the experiments and evaluations carried out to validate and tune the proposed solution’s performance. The experiments will both involve performance evaluation and hyperparameter tuning.

In our experiments we thoroughly evaluate the detecting and tracking stages, individually. Each experiment involves a subset of experiments to test and evaluate different aspects of the stages. The experiments include purpose and procedure to cover why and how an experiment is carried out. This is followed by a description of the presented results and a discussion to explain how to interpret the results.

**Figure 14:**
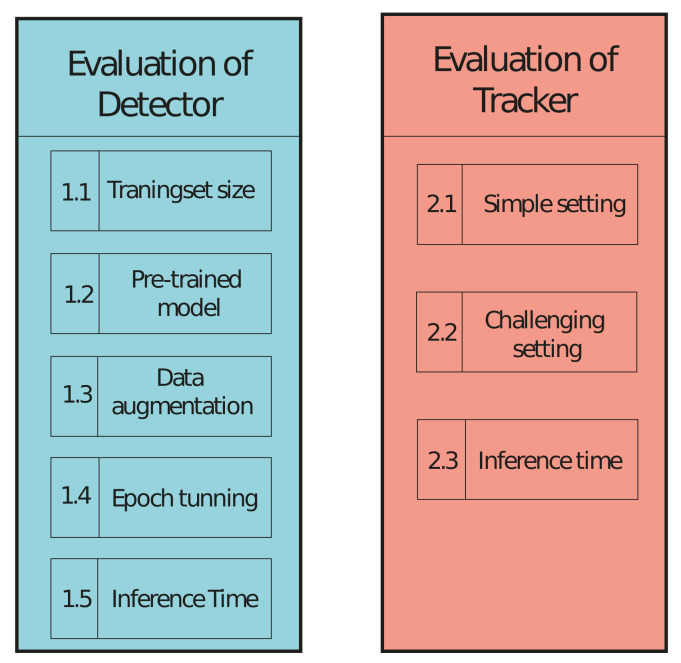
Overview of the different evaluation phases.

On Figure 14 is an overview of the three evaluation phases and the experiments within each evaluation phase. First, the experiment “Training set size” will be carried out, and because it is the first experiment in the evaluation of the detector, it is named experiment 1.1. The following experiment is then named 1.2 and so on. The first number represents the evaluation phase, and the second number represents the experiment number within that evaluation phase.

### 5.1. Benchmark of Model Performance

The following section covers the metric used to evaluate the performance of the proposed solution. Metrics require the experiments to be comparable, measurable, and reproducible. This is important as experiments can potentially involve different data combinations, hyperparameters, and other techniques, making it impossible to compare the predictive performance without a common metric. The system consists of different stages, and as mentioned, these stages are evaluated individually. Thus, not all metrics listed in this section are used to evaluate all parts of the system. For instance, different metrics will be used to evaluate the detector than to evaluate the tracker.

#### 5.1.1. Average Precision (AP)

Percentage of correct positive predictions among all predictions made. It is a measure of how accurate the predictions are in percentage. This is the most frequently used evaluation metric for object detection in recent years. Average precision (AP) is used to evaluate the performance with respect to a specific category. The mean average precision (mAP) is usually used as a final metric for performance evaluation. It is an average of AP for all object categories. The equation for AP is as followed [31]:

(11) $$AP=\frac{TP}{TP+FP}$$

where *TP* is the number of true positives, and *FP* is the number of false positives. This metric will be used to evaluate the prediction accuracy of the detector.

#### 5.1.2. Intersection over Union (IoU)

Intersection over union is used to measure the object localization accuracy by estimating the amount of overlap between two bounding boxes or segmentation masks [31].

The IoU is used the check whether the overlap of a prediction and the ground truth is greater or below a predefined threshold. The AP within the COCO challenge [32] is evaluated with different IoU. The authors calculate for IoU in the range from 50% to 95% with a step size of 5%. This type is usually annotated as AP@50:5:95 and is a popular method to define the IoU threshold. Single IoU are also evaluated where common values as 50% and 75% are used. These are reported as AP50 and AP75. The use of IoU will be seen in both the evaluation of the detector and the tracker.

#### 5.1.3. Multiple Object Tracing Accuracy (MOTA)

Multiple object-tracking accuracy (MOTA) is perhaps the mostly used metric to evaluate a tracker’s performance. It was first introduced in [33], and have since been a CLEAR metrics provided by the CLEAR program which is a program organized to harmonize evaluation task. One reason for MOTA’s popularity is because it uses three sources of errors. It combines the number of false negatives, false positives and id switches compared to the ground truth.

(12) $$MOTA=1-\frac{\sum_{t}\left(FN_{t}+FP_{t}+IDSW_{t}\right)}{\sum_{t}GT_{t}}$$

*FN* is false negatives, *FP* is false positives, *IDSW* is the number of ID switches, *GT* is the ground truth, and *t* is the frame index. *MOTA* results are reported in percentage and can range from [−∞, 100]. The negative value of MOTA appears if the number of errors made by the tracker exceeds the number of all objects in the scene [34]. MOTA will be used in the evaluation of the tracker.

#### 5.1.4. Multiple Object-Tracking Precision (MOTP)

Like multiple object tracking accuracy (MOTA), multiple object tracking precision (MOTP) was first introduced in [33] by the CLEAR program. MOTP aims to obtain a precision score of the tracker. This is done by calculating the average dissimilarity between all true positives and their corresponding ground-truth targets.

(13) $$MOTP=\frac{\sum_{t,i}d_{t,i}}{\sum_{t}c_{t}}$$

where $c_{t}$ is the number of matches in frame t and $d_{t,i}$ is the overlap between target *i* and its assigned ground-truth object. In other words, MOTP gives an average overlap between all correctly matched bounding boxes and their respective ground-truth objects [34]. MOTP will be used in the evaluation of the tracker.

### 5.2. Evaluation of the Detector

This section covers the first phase of the evaluation. This phase is carried out to evaluate the performance of the detector in the proposed solution.

The performance of a detector is strongly connected to the quality of the dataset used for training the detector. If the training set lacks or has very few representations of a specific category, the trained detector model will naturally have difficulty detecting this specific category afterward. A well-balanced and representative dataset of the environment is needed to ensure the best conditions for the model to perform well on the test set. The output from this evaluation phase is an optimal detector model for instance-based semantic segmentation.

The evaluation of the detector will consist of five different experiments. The first experiment will evaluate the model trained on different sizes of datasets. This is to analyze the effect a dataset used for training the model has on the prediction accuracy. Furthermore, obtain the optimal dataset for training the model. The second experiment will test whether the best prediction results are obtained by training a new model from scratch or fine-tuning a pre-trained model. The third experiment will involve the effect of data augmentation. This is to investigate if performing data augmentation on the training data will positively affect the prediction accuracy. The fourth experiment will involve epoch tuning of the model to examine what number of epochs yields the best model accuracy. The fifth and last experiment will examine the inference time of the detector and how the inference time is affected by an increasing number of objects to detect.

### 5.3. Experiment 1.1: Trainingset Size

**Purpose:** Examine if an increase in the size of the dataset used for training the model yields a better prediction accuracy.

**Procedure:** The data used for training is the backbone of any machine-learning algorithm, as a model cannot make any valid predictions without it. Hence, to get the best possible model for detection, it is essential to build a comprehensive, high-quality training dataset. In this experiment, the dataset used for training will be in focus. It will assess in both experiments if an increase of data samples in the training set will improve the overall detection accuracy. Furthermore, test if it is possible to improve the model’s ability to accurately detect the most challenging category by increasing the number of training samples in which this specific category is represented. This experiment aims at determining the optimal dataset used for training the model in terms of mAP for bounding boxes and segmentation.

**Figure 15:**
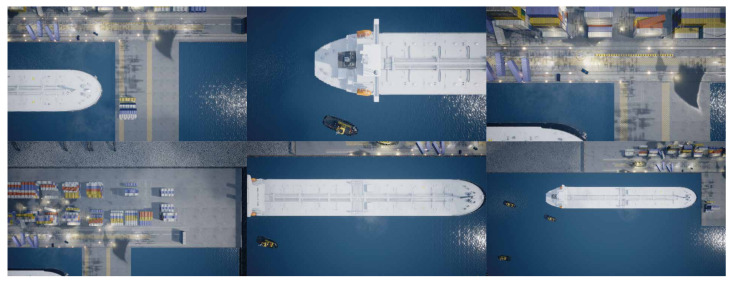
Examples of images in dataset *version 1.1*.

**Figure 16:**
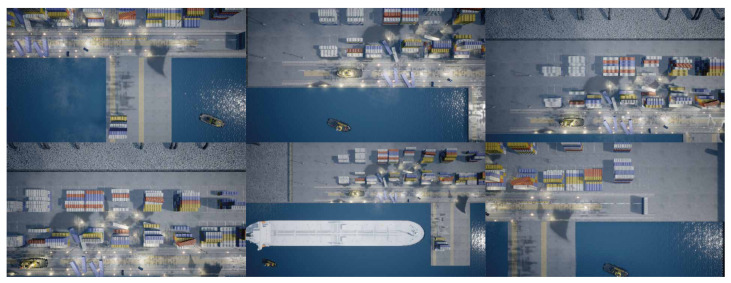
Example of new images in dataset *version 1.2*.

**Results:** Three different versions of datasets used for training are examined in this experiment. The initial training dataset is called *version 1.0* and consists of 882 images from the simulated harbor environment. To test if the prediction accuracy of the model will increase by enlarging the initial dataset, 350 images are added to the *version 1.0* dataset. The new dataset is called *version 1.1*. A snipped of the images added in *version 1.1* can be seen on Figure 15. *Version 1.2* is a further expansion of the *version 1.1* dataset. The added training images mostly consist of the category “land” to improve the model’s ability to detect this category. A snippet of the images added in *version 1.2* can be seen on Figure 16.

**Figure 17:**
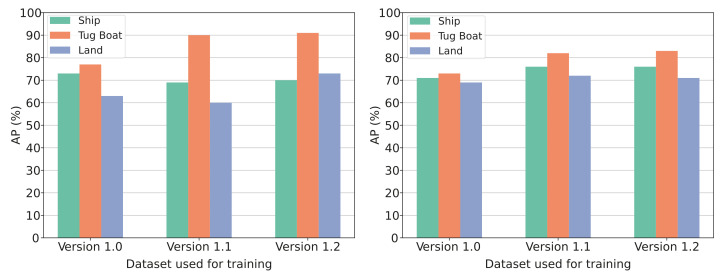
(**Left**) AP for bounding boxes for each category and dataset version. (**Right**) AP for segmentation for each category and dataset version.

**Figure 18:**
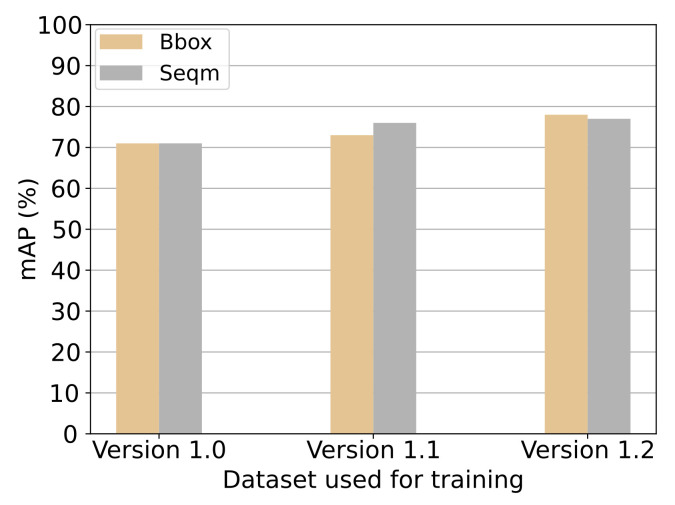
mAP of bounding box and segmentation for each dataset version.

The results from experiment 1 is presented in Figure 17 and Figure 18. The metric used are the AP@50:5:95 explained in Section 5.1. The increase in data samples from dataset *version 1.0* to *version 1.1* showed a small increase in mAP for both bounding-box prediction and segmentation (see Figure 18). From Figure 17 it can be seen that only the category “tugboat” increased for bounding-box accuracy, but all three categories increased in segmentation accuracy. The further expansion of dataset *version 1.1* to *version 1.2* showed a significant increase in bounding-box prediction accuracy for the “land” category (see Figure 17. Additionally, the mAP for bounding boxes increased (see Figure 18).

**Discussion:** From the results presented in Figure 17 and Figure 18 the tendencies in the metrics can be investigated. It can be seen how an increase in image samples used for training the model increases the model’s ability to predict categories correctly. In particular, the increase in training data samples from *version 1.1* to *version 1.2* with a focus on the category “Land” showed that by increasing the number of image samples representing the poorly classified category, the bounding-box accuracy increased significantly. Results presented on Figure 18 show that the model trained on *version 1.2* dataset yielded the best results both in terms of bounding box and segmentation predictions. Therefore, this training dataset will be preferred.

### 5.4. Experiment 1.2: Pre-Trained Model

**Purpose:** Investigate if using a pre-trained model and fine-tune to this thesis dataset will increase the detector’s performance.

**Procedure:** To obtain the best performing model possible different settings when training the model is tested. Usually, fine-tuning a model is a method used when the dataset size is smaller than the number of parameters in the model [35]. The pre-trained backbone network is trained on the COCO train2017 dataset [36]. The box predictor and mask predictor will be trained from scratch. This is compared to training the entire model from scratch.

**Figure 19:**
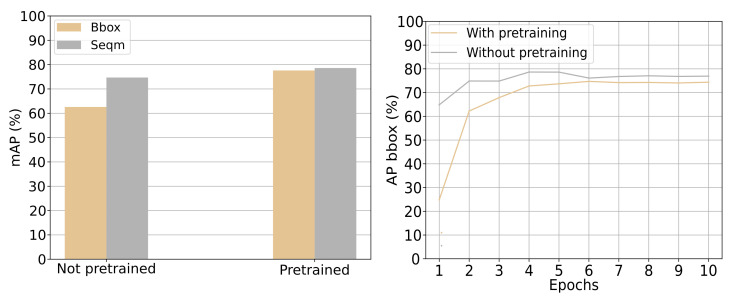
(**Left**) mAP for the best performing model. The best performing model was found based on the plot on the right. Pre-trained model has been trained for 5 epochs, not pre-trained has been trained for 6 epochs. (**Right**) mAP when tested on the test set over epochs.

**Results:** On Figure 19 the left side shows the mAP of the pre-trained model and the model trained from scratch. There is a difference between bounding-box precision of 15%. The difference for segmentation is lower at 4%. On the right side, the mAP for the pre-trained model and the model trained from scratch over epochs can be seen. On Figure 19 both sides show that the pre-trained model has better performance than the model trained from scratch.

**Discussion:** Based on the result on Figure 19 it can be seen that there is a performance gain in fine-tuning the detector model. It might be because the COCO dataset enhances the backbone network to capture universal features such as curves and edges better. Using the pre-trained backbone network ensures no overfitting to the training data, which also explains the worse performance of the model without pre-training. Based on the results on Figure 19 the pre-trained variant of the detector was chosen as the preferred model.

### 5.5. Experiment 1.3: Data Augmentation

**Purpose:** Examine if there is a performance boost in average precision when adding data augmentation to the training set.

**Procedure:** As explained in Section 3.1, the training set contains only simulated data which can have a negative impact on the performance. Data augmentation can be used to make the images in the training set less similar. Data augmentation means changing some visual aspect of an image (e.g., changing color hue, brightness, saturation, or rotating the image). In this experiment, five different data augmentation settings will be tested see Table 1. The augmentation performed on the training data will be applied to 50% of the data samples picked randomly. The experiment aims at determining the performance gains using data augmentation while also trying different settings to find the best possible performance gains.

**Figure 20:**
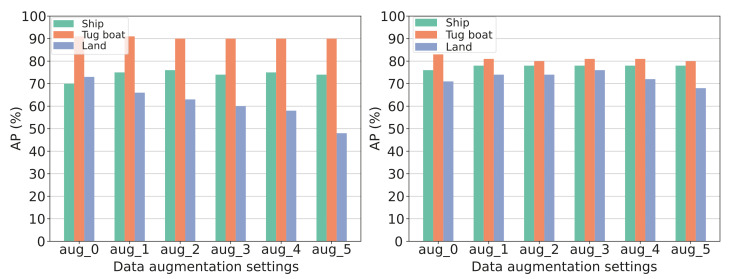
Average precision of categories individually with different levels of data augmentation enabled. (**Left**) AP for bounding boxes. (**Right**) AP for segmentation.

**Figure 21:**
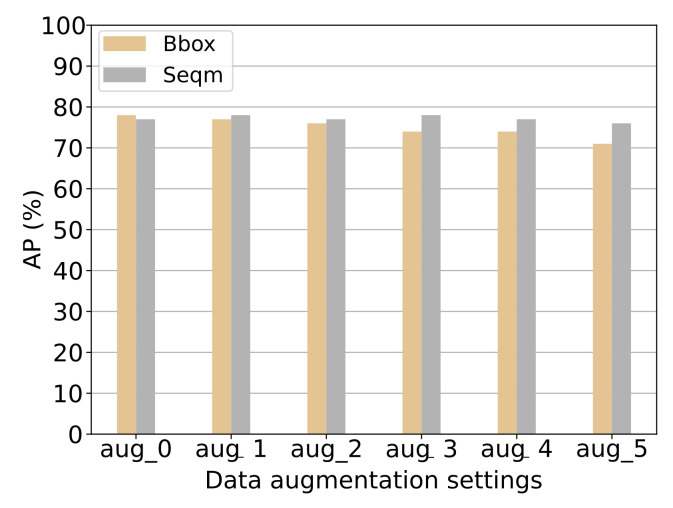
mAP with different levels of data augmentation used.

**Results:** The results from the experiment can be seen in Figure 20 and Figure 21. Figure 20 presents the AP@50:5:95 results of both bounding box and segmentation for each category individually, with each grouped bar representing a different level of data augmentation. Figure 21 presents the mAP results for both bounding box and segmentation, with each grouping representing a different level of data augmentation. The results show how the data augmentation gives a minor increase in the model’s ability to predict bounding boxes for the category “large vessel” accurately but, simultaneously, a significant decrease in its ability to predict the bounding boxes for the category “land” accurately. The category “tugboat” seems to be unaffected by the data augmentation. The models’ ability to accurately segment the categories increase a little for both “large vessel” and “land” until augmentation setting aug_3. From Figure 21 it can be seen that the model’s overall ability to detect bounding boxes correctly decreases with the increasing amount of augmentation applied. The segmentation seems unaffected, with a minor increase in augmentation setting aug_1 and aug_3.

**Discussion:** Data augmentation should, in theory, give a noticeable performance boost. However, this is not the case in this experiment. As can be seen on Figure 21 the result becomes worse for predicting bounding boxes as more data augmentation is enabled. One explanation is that the test set is also made from simulated data with the same assets. The augmentation makes the training data less similar to the test set, giving a lower AP. This similarities in the test and training domain makes the detection network favor overfitting the data instead of learning a generalised description of the vessels. It makes sense to use data augmentation when the model is trained for real-world environments. However, as of now, the system will only be applied to simulated data. No data augmentation will be enabled for training.

### 5.6. Experiment 1.4: Epoch Tuning

**Purpose:** Evaluating what number of epochs yields the best model performance.

**Procedure:** When training a machine-learning model for detection, the model converges towards values of weights for which the loss is minimum, also called the minima. Nevertheless, it is often not interesting or satisfying to reach the minima in training data loss, as this can result in overfitting the model towards the training data. Instead, it is interesting to investigate the number of iterations needed to reach the highest accuracy on the test data. This best reflects how the model performs on data on which it was not trained. This experiment aims at determining the optimal number of iterations needed to reach the highest accuracy on the test data. The optimal number of iterations is found when the mAP on the test set does not increase anymore.

**Figure 22:**
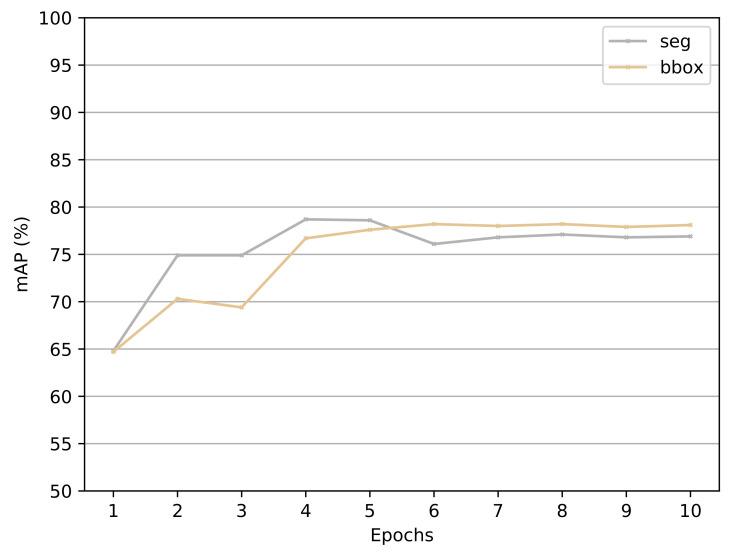
mAP with different levels of data augmentation used over epochs.

**Results:** The results are presented on Figure 22. With an increasing number of epochs, the mAP is increasing significantly until 4 iterations. From epoch 4 to 5, the mAP for bounding boxes is still increasing. The subsequent increase from 5 to 10 does not yield higher accuracy.

**Discussion:** With no significant increase in mAP on the test set after 5 epochs, yielding only a slight increase in mAP for bounding and a decrease in mAP for segmentation. The experiment could have been extended to test even more epochs, but the mAP flattened after 7 epochs, with only the training time continuing to increase. From the results, 5 epochs were chosen as the optimal number of epochs.

### 5.7. Experiment 1.5: Inference Time

**Purpose:** To evaluate how the inference time is affected by the number of objects to detect.

**Procedure:** In this experiment, the inference time of the object detection stage will be tested. The object detection stage runs on an RTX 2060 GPU, an AMD Ryzen 5600 and 16 GB of RAM. The detector will be tested by detecting objects in 8 different video sequences. The first sequence will only involve one vessel, then two vessels to detect, then three, then four, five, 10, 20, and at last 40 vessels to detect. This experiment aims to investigate how the number of vessels affects the inference time of the detector stage.

**Figure 23:**
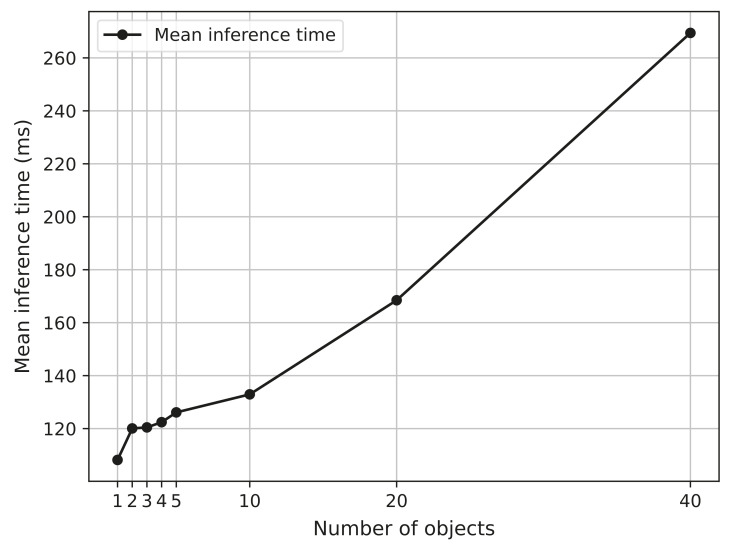
Mean inference time for the detection stage over number of objects.

**Results:** The results from this experiment can be seen on Figure 23. The experiments show a penalty on inference time when adding more vessels to an image. In the best case of only having a single vessel in the image, the inference time is around 110 ms. When looking at the worst case at 40 objects, the mean inference time has increased to just over 260 ms.

**Discussion:** The results clearly show that as the number of vessels increases, the mean inference time increases. The increase in inference seems to have a close to linear relationship with the number of objects. The increase in inference might be caused by the extra calculations needed for rotating multiple bounding boxes. Furthermore, an increase in vessels will yield more deleted bounding boxes needed to be done by NMS. This could be part of the reason for the increase in inference time. The increase in inference time is quite significant and should in the future be lowered for this system to be able to run at real-time. It is unlikely that there would be 40 vessels in the image at one time, so this can be considered a worst-case scenario.

### 5.8. Summary

The evaluation of the detector stage showcases how different training methods yield the best result for the detector in this project. It is essential to obtain the best possible detector model as it is the first building block of the proposed solution, which the two other stages build upon. The first experiments show that an increase in the dataset used for training increases the detector’s accuracy for both bounding-box localization and pixel-level segmentation. As a result, the most extensive training set is used in the following experiments. The second experiment tested whether a pre-trained backbone network on the COCO 2017 dataset would yield a better result than a backbone network trained from scratch. This experiment showed significantly better results when the pre-trained model was applied, and this configuration is used in future experiments. In the third experiment, data augmentation is in focus. Five different configurations for data augmentation on the training set are used. The results showed no significant improvements in the use of data augmentation. Hence data augmentation is not used in the following experiments. The fourth experiment aims to find the optimal number of training iterations to achieve the best model performance. The result showed that a total number of 5 epochs resulted in the best model performance. The last experiment of the detector evaluates the inference time and how it is affected by an increased number of objects to detect. The experiment showed a close to linear relationship between inference time and number of objects to detect.

### 5.9. Evaluation of the MOT Stage

This section deals with the second phase of the evaluation. The experiments in this phase are carried out to evaluate the MOT stage of the system, in terms of accuracy, precision, and inference time but also its limitations.

The evaluation of the MOT stage will consist of three experiments. The first experiment evaluates the performance of the tracker in a simple scene. These results will serve as a basis for how well the tracker performs if no challenging settings are applied. The second experiment will investigate the performance of the tracker in a more challenging setting. Here, challenges such as occlusions and dynamic movement of the camera’s viewpoint are applied. The third and final experiment will evaluate the efficiency of the tracker to test how the inference time is affected by an increasing number of objects to track.

For the Kalman filter used in the deep sort tracking methodology, we adopted a constant velocity model. The 8 × 8 process noise covariance matrix parameters were considered static and were set to

(14) $$\sigma_{x}=\sigma_{y}=\sigma_{h}=\frac{1}{20};\sigma_{\gamma =}=\frac{1}{100}$$

(15) $$\sigma_{\dot{x}}=\sigma_{\dot{y}}=\sigma_{\dot{h}}=\frac{1}{160};\sigma_{\dot{\gamma }}=\frac{1}{10,000}$$

for the position and velocity components, respectively. The 4 × 4 measurement noise covariance matrix parameters were set to

(16) $$\sigma_{x}=\sigma_{y}=\sigma_{h}=\frac{1}{20};\sigma_{\gamma }=\frac{1}{100}$$

throughout all our experiments.

### 5.10. Experiment 2.1: Simple Setting

**Purpose:** Evaluate the tracker in simple scenes where no challenging features are applied.

**Procedure:** The experiment consists of two scenarios, each involving three sequences. Both scenarios take place in a simulated harbor environment and involve a UAV hovering in a static position without any objects occluding the camera attached to the UAV at any point. The three sequences in the first scenario involve both moving and static objects to track. The three sequences in the second scenario involve only moving objects to track. These results will act as a baseline for later experiments where the tracker will be tested in more challenging scenarios.

**Results:** The quantitative results derived from the tracker tested on scenario 1 and 2 is presented from Table 2, Table 3, Table 4 and Table 5. The results from scenario 1 show a perfect score in MOTA for category 1 (large vessel) in all three sequences, resulting in 0 false negatives, 0 false positives, and 0 ID switches. Category 2 (tugboat) performs almost a perfect score in MOTA, with a combined result of 98.15%. Both categories perform very well on the MOTP metric with respective results of 95.07 and 93.47. The results from scenario 2 show an almost perfect score in MOTA for both categories caused by the tracker’s ability to perform good with few false positives, false negatives, and ID switches. The MOTP for decreases for both category 1 and 2 in scenario 2.

**Discussion:** From the quantitative results, the tracker show almost performs perfect tracking results on both categories in the ideal settings created in scenarios 1 and 2. A minor decrease in the MOTA result for both categories shows the tracker does an accurate job predicting objects correctly in an ideal setting.

As mentioned, the MOTP metric result decreases for both categories from scenario 1 to scenario 2. This is expected as all objects in scenario 2 move, making it harder for the tracker to correctly predict the placement of all bounding boxes.

These results showcase how well the tracker performs in the ideal setting where the camera’s viewpoint is static and no occlusions disturb the camera’s viewpoint.

### 5.11. Experiment 2.2: Tracker Performance-Challenging

**Purpose:** Evaluate the tracker in challenging settings where occlusion and movement are applied to the camera’s viewpoint by moving the UVA.

**Procedure:** Two new scenarios have been constructed, called scenarios 3 and 4, to evaluate the tracker’s performance in challenging settings. Both scenarios involve three sequences each. All sequences take place in a simulated harbor environment. The three sequences in scenario 3 involve the UAV hovering at a fixed point. Occlusions are applied to disturb the camera’s viewpoint to test how this affects the tracker’s performance. The three sequences in scenario 4 involve the UAV flying around to dynamically changing the camera’s viewpoint. The aim of this experiment is to the evaluate how the tracker performs under challenging settings. A challenging setting such as occlusion and movement of the camera’s viewpoint are known challenges for a MOT algorithm. Therefore, we generated simulations with occlusions and camera motion due to UAV movement.

**Results:** The results are presented from Table 6, Table 7, Table 8 and Table 9. The results from scenario 3 show a decrease in MOTA for both classes compared to the results from scenario 2 see Table 4 and Table 5, this is caused by a significant increase in false negatives, false positives, and ID switches. On the other hand, the MOTP metric results did not show a remarkable decrease in the precision. Results from scenario 4 shows almost unchanged results for category 1 for both MOTA and MOTP compared to results from Table 4 and Table 5. The results for category 2 shows a small decrease in both MOTA and MOTP.

**Discussion:** The results for scenario 3 seen on Table 6 and Table 7 shows occlusions of the camera’s viewpoint causes the tracker to perform a significant increase in false negative, false positive, and ID switches hence the significant decrease on MOTA. The decrease in MOTA is expected as occlusions is a complex challenge for tracking algorithms to solve. MOTP is kept considerably constant, which is also expected as occlusions should not affect the trackers’ ability to locate bounding boxes precisely. Results from scenario 4 seen on Table 8 and Table 9 show good results for category 1. This shows that the tracker is good at tracking category 1 even if the camera’s viewpoint moves. The results for category 2 see Table 8 and Table 9 show a decrease in both MOTA and MOTP, this is caused by category 2 objects leaves and re-enters the scene during the 3 sequences. Objects leaving and re-entering the scene is a complex task for tracking algorithms to handle, causing the tracker to increase ID switches. The increasing number of false positives and false negatives can be explained by an object located on the edge of an image, making it hard for the tracker to detect the objects correctly.

### 5.12. Experiment 2.3: Tracker Performance—Inference Time

**Purpose:** To evaluate how the inference time is affected by the number of objects to track.

**Procedure:** An Nvidia RTX 2060 GPU, an AMD Ryzen 5600× CPU with 16 GB of RAM, is used for these experiments. Please note that it is not possible to test the MOT stage by itself as it needs a detector to feed it with bounding-box predictions. However, it is still possible to test the inference time for the MOT stage itself. The experiment aims to investigate how the number of objects to track affects the inference time of the MOT stage.

**Figure 24:**
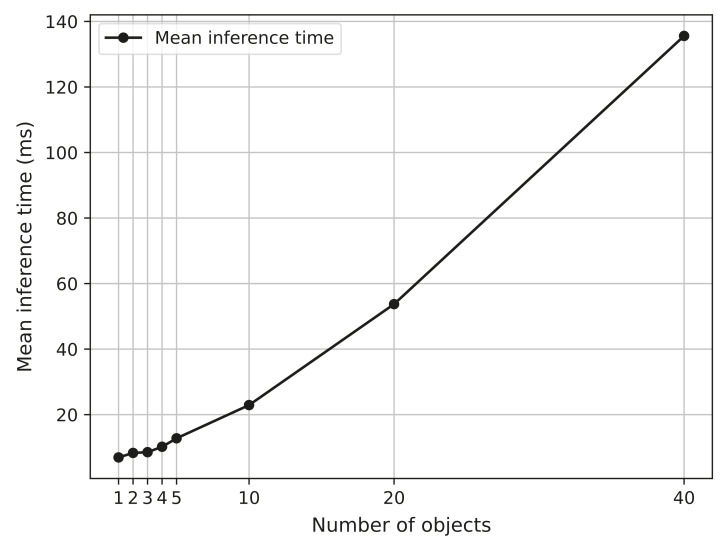
Mean inference time for MOT stage over number of objects.

**Results:** On Figure 24 the inference time for the tracker can be seen. The graph shows an increase in inference time when more vessels are introduced. The best-case results are when there is one object in the image, and the worst case is when there are 40.

**Discussion:** There is a decrease in efficiency with more vessels to track. The results on Figure 24 show a slight exponential increasing tendency when more vessels are introduced to the test. However, even at 40 vessels, the inference is comparable to that of the detector stage performed at five vessels, which alludes to the detector stage being a bottleneck in making this system appropriate for real-time applications. The exponential increase is believed to stem from both computing metrics for all tracks and the Hungarian assignment algorithm inside Deep SORT.

### 5.13. Summary

The evaluation of the MOT stage aims to evaluate the performance of the tracker in different scenarios quantitatively. The first experiment tests the tracker in simple scenes, where no occlusions or movement of the UAV during the video sequence is applied. Here show the tracker almost perfect scores on both categories. In the second experiment, the tracker is tested by more challenging scenes. Both occlusions and movement of the UVA during the video sequences were applied. The results show that the tracker still scores high in both MOTA and MOTP but find occlusions more difficult, yielding in an increased number of ID switches. On Table 10 is a combined table of all four scenarios across object categories shown.

In the third and last experiment, the inference time of the tracker is tested to see how the inference time is affected by an increasing number of objects to track. The results show a slight exponential increasing tendency when more vessels are introduced to the test.

## 6. Conclusions

In this work we proposed a complete solution for multiple object detection and tracking of maritime vehicles from aerial monocular images, using deep appearance features. A set of experiments in a realistic simulation environment validate the applicability of our system.

The proposed system is not restricted to be used in harbor environments. It could also provide large vessels with assistance when sailing through critical and narrow passages.

Furthermore, even though the specific system is developed to work in a maritime environment, it can be applied in other contexts, including but not limited to traffic monitoring and time to collision estimation, as long as domain-specific training data are provided and the shape of the tracked objects can be represented by a bound box without too much overhead.

Finally, our proposed tracking approach currently does not consider self-motion of the camera (i.e., the UAV is assumed statically hovering). In the future one could easily extend the system dynamics and observation models to incorporate inertial and absolute measurements (e.g., GPS, IMU, altimeter) from the UAV platform, to allow the system to deal with self-motion. 

## Figures and Tables

**Table 1:** Table of notations used for data augmentation with different setting enabled.

Data Augmentation Setting	Data Augmentation
aug_0	No data augmentation
aug_1	Horizontal flip
aug_2	Horizontal flip + Brightness
aug_3	Horizontal flip + Brightness + Contrast
aug_4	Horizontal flip + Brightness + Contrast + Color saturation
aug_5	Horizontal flip + Brightness + Contrast + Color saturation + Color hue

**Table 2:** **Scenario 1—category 1** Quantitative results of the tracker’s performance to track objects of category 1 (large vessel) in scenario 1. Scenario 1 consists of three sequences involving both moving and static objects, and no occlusion or dynamic movement of the camera is applied.

Sequence No.	MOTA	MOTP	TP	FN	FP	IDSW
1	100	95.55	423	0	0	0
2	100	94.85	406	0	0	0
3	100	94.77	372	0	0	0
**Combined**	**100**	**95.07**	**1201**	**0**	**0**	**0**

**Table 3:** **Scenario 1—category 2** Quantitative results of the tracker’s performance to track objects of category 2 (tugboat) in scenario 1. Scenario 1 consists of three sequences involving both moving and static objects, and no occlusion or dynamic movement of the camera is applied.

Sequence No.	MOTA	MOTP	TP	FN	FP	IDSW
1	96.74	93.28	1238	19	18	4
2	98.15	95.20	1596	28	2	0
3	99.33	91.76	1480	8	2	0
**Combined**	**98.15**	**93.47**	**4314**	**55**	**22**	**4**

**Table 4:** **Scenario 2—category 1** Quantitative results of the tracker’s performance to track objects of category 1 (large vessel) in scenario 1. Scenario 1 consists of three sequences involving only moving objects, and no occlusion or dynamic movement of the camera is applied.

Sequence No.	MOTA	MOTP	TP	FN	FP	IDSW
1	100	95.83	440	0	0	0
2	96.96	90.10	391	4	7	1
3	100	88.24	291	0	0	0
**Combined**	**98.93**	**91.86**	**1122**	**4**	**7**	**1**

**Table 5:** **Scenario 2—category 2** Quantitative results of the tracker’s performance to track objects of category 2 (tugboat) in scenario 1. Scenario 1 consists of three sequences involving only moving objects, and no occlusion or dynamic movement of the camera is applied.

Sequence No.	MOTA	MOTP	TP	FN	FP	IDSW
1	96.29	90.54	1877	34	31	6
2	96.78	90.06	1594	23	20	9
3	97.20	89.00	1465	8	4	5
**Combined**	**97.20**	**89.93**	**4936**	**65**	**55**	**20**

**Table 6:** **Scenario 3—category 1** Quantitative results of the tracker’s performance to track objects of category 1 (large vessel) in scenario 3. Scenario 3 consists of three sequences involving both moving and static objects, occlusions of the camera’s viewpoint are applied.

Sequence No.	MOTA	MOTP	TP	FN	FP	IDSW
1	62.50	88.67	86	18	18	3
2	68.79	89.11	297	33	48	22
3	64.27	87.63	372	40	63	31
**Combined**	**65.88**	**88.37**	**718**	**91**	**129**	**56**

**Table 7:** **Scenario 3—category 2** Quantitative results of the tracker’s performance to track objects of category 2 (tugboat) in scenario 3. Scenario 3 consists of three sequences involving both moving and static objects, occlusions of the camera’s viewpoint are applied.

Sequence No.	MOTA	MOTP	TP	FN	FP	IDSW
1	76.20	89.00	455	3	67	39
2	77.19	87.94	1519	99	164	106
3	75.9	88.19	1696	109	176	150
**Combined**	**76.48**	**88.19**	**3670**	**211**	**407**	**295**

**Table 8:** **Scenario 4—category 1** Quantitative results of the tracker’s performance to track objects of category 1 (large vessel) in scenario 3. Scenario 3 consists of three sequences involving both moving and static objects, dynamic movement of the camera’s viewpoint are applied.

Sequence No.	MOTA	MOTP	TP	FN	FP	IDSW
1	100	88.77	188	0	0	0
2	93.21	90.92	357	26	0	0
3	98.49	87.84	264	0	2	2
**Combined**	**96.41**	**89.41**	**809**	**26**	**2**	**2**

**Table 9:** **Scenario 4—category 2** Quantitative results of the tracker’s performance to track objects of category 2 (tugboat) in scenario 3. Scenario 3 consists of three sequences involving both moving and static objects, dynamic movement of the camera’s viewpoint are applied.

Sequence No.	MOTA	MOTP	TP	FN	FP	IDSW
1	80.06	83.30	606	61	41	31
2	90.48	86.59	1301	65	41	24
3	82.63	85.49	425	47	27	8
**Combined**	**86.23**	**85.54**	**2332**	**173**	**109**	**63**

**Table 10:** This table pretenses a weighted average sum of results conducted from experiments 1 and 2. It shows the overall performance of the tracker across scenarios and categories.

Scenario No.	MOTA	MOTP	TP	FN	FP	IDSW
1	98.55	93.82	5515	55	22	4
2	97.52	90.29	6058	69	62	21
3	74.65	88.22	4388	302	536	351
4	89.00	86.59	3141	199	111	65
**Combined**	**90.89**	**87.98**	**19,102**	**625**	**497**	**441**

## Data Availability

Not applicable.
